# The Role of DNA Repair (*XPC*, *XPD*, *XPF*, and *XPG)* Gene Polymorphisms in the Development of Myeloproliferative Neoplasms

**DOI:** 10.3390/medicina60030506

**Published:** 2024-03-19

**Authors:** Adriana-Stela Crișan, Florin Tripon, Alina Bogliș, George-Andrei Crauciuc, Adrian P. Trifa, Erzsébet Lázár, Ioan Macarie, Manuela Rozalia Gabor, Claudia Bănescu

**Affiliations:** 1 Genetics Department, ‘George Emil Palade’ University of Medicine, Pharmacy, Science, and Technology of Targu Mures, Gheorghe Marinescu 38, 540142 Targu Mures, Romania; adriana.cosma17@gmail.com (A.-S.C.); tripon.florin.2010@gmail.com (F.T.); alinaboglis@gmail.com (A.B.); andrei.crauciuc@gmail.com (G.-A.C.); 2 Genetics Laboratory, Center for Advanced Medical and Pharmaceutical Research, ‘George Emil Palade’ University of Medicine, Pharmacy, Science, and Technology of Targu Mures, Gheorghe Marinescu 38, 540139 Targu Mures, Romania; 3 Department of Genetics, ‘Victor Babeș’ University of Medicine and Pharmacy, 300041 Timisoara, Romania; trifa.adrian@gmail.com; 4 Department of Internal Medicine, ‘George Emil Palade’ University of Medicine, Pharmacy, Science, and Technology, 540142 Targu Mures, Romania; erzsebetlazarbenedek@gmail.com (E.L.); ioan.macarie@umfst.ro (I.M.); 5 Department of Economic Sciences, ‘George Emil Palade’ University of Medicine, Pharmacy, Science, and Technology, 540136 Targu Mures, Romania; manuela.gabor@umfst.ro

**Keywords:** myeloproliferative neoplasms, NER, *XPC*, *XPD*, *XPF*, *XPG*, gene polymorphism

## Abstract

*Background and Objectives*: Several polymorphisms have been described in various DNA repair genes. Nucleotide excision DNA repair (NER) detects defects of DNA molecules and corrects them to restore genome integrity. We hypothesized that the *XPC*, *XPD*, *XPF*, and *XPG* gene polymorphisms influence the appearance of myeloproliferative neoplasms (MPNs). *Materials and Methods*: We investigated the *XPC* 1496C>T (rs2228000, *XPC* Ala499Val), *XPC* 2920A>C (rs228001, *XPC* Lys939Gln), *XPD* 2251A>C (rs13181, *XPD* Lys751Gln), *XPF*-673C>T (rs3136038), *XPF* 11985A>G (rs254942), and *XPG* 3507G>C (rs17655, *XPG* Asp1104His) polymorphisms by polymerase chain reaction–restriction fragment length polymorphism analysis in 393 MPN patients [153 with polycythemia vera (PV), 201 with essential thrombocythemia (ET), and 39 with primary myelofibrosis (PMF)] and 323 healthy controls. *Results*: Overall, we found that variant genotypes of *XPD* 2251A>C were associated with an increased risk of MPN (OR = 1.54, 95% CI = 1.15–2.08, *p* = 0.004), while *XPF*-673C>T and *XPF* 11985A>G were associated with a decreased risk of developing MPN (OR = 0.56, 95% CI = 0.42–0.76, *p* < 0.001; and OR = 0.26, 95% CI = 0.19–0.37, *p* < 0.001, respectively). *Conclusions*: In light of our findings, *XPD* 2251A>C polymorphism was associated with the risk of developing MPN and *XPF*-673C>T and *XPF* 11985A>G single nucleotide polymorphisms (SNPs) may have a protective role for MPN, while *XPC* 1496C>T, *XPC* 2920A>C, and *XPG* 3507G>C polymorphisms do not represent risk factors in MPN development.

## 1. Introduction

Myeloproliferative neoplasms (MPNs) constitute a category of clonal malignancies that may lead to the overproduction of terminally differentiated cells of one or more elements of the myeloid lineage [1,2,3]. Polycythemia vera (PV), essential thrombocythemia (ET), and primary myelofibrosis (PMF), due to their clinical, morphological, and molecular features, are organized into Philadelphia-negative classical MPNs or *BCR-ABL*-negative classical MPNs [4,5,6]; they are distinguished by extramedullary hematopoiesis and a predisposition for fibrosis, hemorrhage, arterial and venous thrombosis, and the possibility to change into acute leukemia [7]. *JAK2* (Janus kinase 2; located on chromosome 9p24), *MPL* (myeloproliferative leukemia virus oncogene; located on chromosome 1p34), and *CALR* (calreticulin; located on chromosome 19p13.2) are specific somatic driver mutations that have been described in the major part of *BCR-ABL*–negative neoplasms [6,8]. The WHO (World Health Organization) diagnostic criteria for MPNs include the driver mutations; therefore in PV the *JAK2* mutation frequency is 98%; in ET the *JAK2*, *CALR*, and *MPL* mutation frequency is 60%, 22%, and 3%, while in PMF the frequency of *JAK2*, *CALR*, and *MPL* mutation is 58%, 25%, and 7% [8]. Some exceptions have been reported, even though *CALR* and *MPL* mutations are normally absent in PV [6,8]. Approximately 10–15% of subjects with ET or PMF do not express any of these mutations and are called “triple-negative” [8,9].

Endogenous and exogenous sources generate constant genotoxic pressure on cells. Every day, a single human cell is subjected to tens of thousands of DNA lesions. These defects should be repaired to avoid chromosomal breakage, blocked replication, and harmful mutations. DNA repair represents a multitude of ways through which living cells can detect alterations in their DNA molecules and correct the damage to reestablish the integrity of their genome. Also, DNA repair can impede the transformation of preneoplastic cells into malignant cells [10] and plays a decisive part in defending cells against ultraviolet (UV) rays, smoking, diet, and ionizing radiation [11]. Initially, the significance of DNA repair in cancer was demonstrated in a study of subjects with xeroderma pigmentosum (XP), characterized by excessive sensitivity to UV rays [10] and by an increased risk of developing melanoma and squamous cell carcinoma when exposed to sunlight [12]. One of the most important DNA pathways is represented by nucleotide excision DNA repair (NER). NER is capable of identifying the DNA damage and removing the chemically and structurally different helix-distorting DNA lesions [13,14]. Seven proteins are considered the main participants of NER and make up the Xeroderma pigmentosum complementary group [15].

The human *XPC* gene is found in chromosome 3p25, comprises 16 exons and 15 introns, and codifies a protein—xeroderma pigmentosum complementation group C (XPC) [16], which is a significant DNA lesion recognition protein involved in NER [17]. The most commonly studied polymorphisms of the *XPC* gene are Ala499Val and Lys939Gln.

The *XPC* Ala499Val (1496C>T, rs2228000) gene polymorphism, with a C to T substitution in exon 8, gives rise to an Ala with Val substitution at position 499 [18]. Some researchers have shown that *XPC* 1496C>T is associated with the risk of breast cancer [19,20] and bladder cancer [21,22,23]. Contradictory results have been reported for hematological diseases. *XPC* 1496C>T has been associated with an increased risk of developing Hodgkin’s Lymphoma [24,25] but was not associated with leukemic risk in patients with PV and ET [26].

*XPC* Lys939Gln (2920A>C, rs2228001) is the most studied single nucleotide polymorphism (SNP) of the *XPC* gene, and there is an exchange at codon 939 from lysine to glutamine [27]. This SNP has been associated with a high risk of different malignant disorders, such as melanoma, lung, colorectal, bladder [18,23], ovarian cancers [28], leukemia [27], and Hodgkin’s Lymphoma [24], but not with acute myeloid leukemia (AML) [29] and leukemic transformation in patients with PV, ET [26].

Excision repair cross-complementation group 2 (*ERCC2*) is well known as *XPD* and is located at chromosome 19q13.3 [30]. The *XPD* gene codifies a DNA helicase implicated in the NER system. Protein function and cellular responses to precise types of DNA damage are affected by *XPD* Lys751Gln (2251A>C, rs13181) polymorphism [31], which is one of the most widely studied polymorphisms of *XPD*. There is a change at codon 751 in exon 23 from lysine to glutamine [30]. *XPD* 2251A>C polymorphism contributes to hematological neoplasms, such as chronic myeloid leukemia (CML) [32,33], AML [29], and AML transformation [26], and some showed no association [34,35,36].

The complex formed between Xeroderma pigmentosum group F (*XPF*) and *ERCC1* (excision repair cross complementation 1) excise the damaged DNA. The susceptibility for different malignancies is influenced by the *XPF* genetic variant [37]. *ERCC5*/*XPG* is found on chromosome 13q22–33 and is constituted by 14 introns and 15 exons. Its protein outcome plays a fundamental part in the NER system [38].

*XPG* Asp1104His (3507G>C, rs17655) includes a substitution of G with C in codon 1104 (leading to an amino acid change from aspartic acid to histidine), which may influence the DNA repair success [39]. Numerous studies have been conducted to investigate the association between *XPG* 3507G>C polymorphism and the risk of multiple cancers [38,40,41,42], and important discrepancies have been reported.

The selected variants *XPC* 1496C>T, *XPC* 2920A>C, *XPD* 2251A>C, *XPF*-673C>T, *XPF* 11985A>G, and *XPG* 3507G>C were studied in different populations for multiple types of cancers: breast cancer [19,20], bladder cancer [18,21,22,23], ovarian cancer [28], hematological diseases such as Hodgkin’s Lymphoma [24,25], PV and ET [26], AML [29], and CML [32,33]. We aimed to evaluate the influence of the DNA repair gene in the occurrence of myeloproliferative neoplasms. We also wanted to establish the association between the studied polymorphisms of the *XPC*, *XPD*, *XPF*, and *XPG* genes and the *JAK2*, *CALR* driver mutations and to identify possible predictors in the appearance of myeloproliferative neoplasms.

## 2. Materials and Methods

### 2.1. Research Ethics Considerations

A case–control study was conducted between 2019 and 2022 following the Declaration of Helsinki after obtaining ‘George Emil Palade’ University of Medicine, Pharmacy, Science and Technology of Targu Mures ethics committee approval (No. 504 from 15 November 2019 and No. 1252 from 28 January 2021). Written informed consent concerning the genetic testing was obtained from each study participant.

### 2.2. Patients and Controls

The present study enrolled 393 unrelated patients diagnosed with MPN according to the latest WHO classification of myeloid neoplasms [43]. The subjects were recruited from the Hematology Clinics in Targu Mures, Romania.

The estimated incidences for PV, ET, and PMF typically range as follows: 0.5 to 2.5 cases; 1.0 to 2.5 cases; and 0.1 to 1.0 cases per 100,000 population per year in Europe. The patients and controls included in the study were from the central region of the country, with an estimated adult population (>20 years old) of 1,764,765 people, according to the National Institute of Public Health, Romania, in 2021 [44].

The sample size for our study was estimated a priori through power analysis by using SPSS 23.0 (licensed) software. This analysis allowed us to determine the total sample size based on a significance level (alpha) set at 0.05 and a test power level of 80% at an effect size of 1.5. The sample size was estimated to be 696 subjects.

The control group included 323 healthy unrelated individuals without known malignancies chosen taking into account the gender and age of the patients. The subjects (patients and controls) were Caucasians from the central region of Romania. The clinical and hematological characteristics of the MPN patients were obtained from clinical records, as well as data related to the treatment. The mean age was 57.76 ± 14.43 years (range 17–85) for patients and 56.15 ± 15.3 years (range 25–94) for controls. There were no significant differences between the two groups regarding gender and age distribution (Table 1). Also, we investigated the constitutional symptoms and venous and arterial thrombotic events in MPN cases included in the present study. By constitutional symptoms, we mean unexplained fever, excessive sweating, fatigue, weight loss, and early satiety. Venous thrombotic events included cerebral sinus vein thrombosis, pulmonary embolism, deep vein thrombosis, and portal or mesenteric vein thrombosis. Arterial thrombotic events included unstable angina pectoris, acute myocardial infarction, transient ischemic attack, ischemic stroke, and peripheral arterial disease.

Regarding treatment, most patients received hydroxyurea (HU), and a small proportion received other cytotoxic agents, anagrelide, or interferon (IFN). Patients who received only HU and other cytotoxic agents were included in the “agents alone or in combination” group, and those who received anagrelide or interferon were included in the “no exposure” group because these drugs are considered non-leukemogenic [26].

### 2.3. SNP Selection

NER may identify and eliminate changes in DNA structure. SNPs of the genes in-volved in the NER may generate differences in DNA repair ability between peoples, and thereby they may affect the susceptibility to MPN. Therefore, SNPs in this research were selected according to their inadequate DNA repair capacity in the NER pathway and the risk allele frequency >0.05 in the European population [45].

The selection criteria of investigated SNPs included a variant allele frequency higher than 0.05 and also considered the reported association with different types of malignancies.

The highest population Minor Allele Frequencies (MAFs) for the SNPs investigated were as follows: *XPC* 1496C>T (rs2228000, MAF—0.48), *XPC* 2920A>C (rs2228001, MAF—0.49), *XPD* 2251A>C (rs13181, MAF—0.45), *XPF*-673C>T (rs3136038, MAF—0.49), *XPF* 11985A>G (rs254942, MAF—0.25), and *XPG* 3507G>C (rs17655, MAF—0.5).

The allele frequency in all populations and in the European population, as well as the most severe consequence and clinical significance of these SNPs, are presented in Table 2.

### 2.4. Sample Collection and Processing

**Figure 1:**
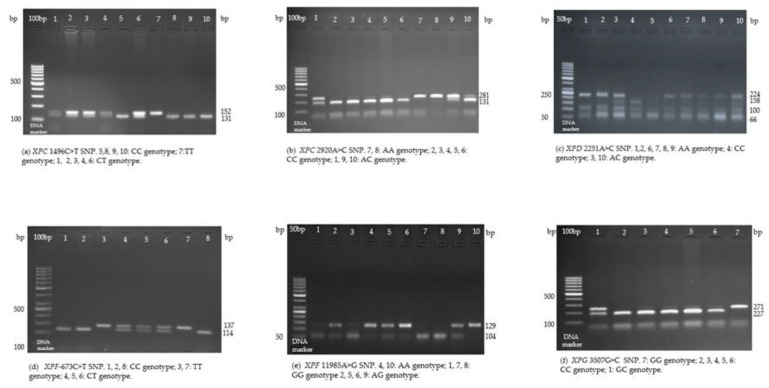
The electropherograms of the *XPC* 1496C>T (rs2228000), *XPC* 2920A>C (rs2228001), *XPD* 2251A>C (rs13181), *XPF*-673C>T (rs3136038), *XPF* 11985A>G (rs254942), and *XPG* 3507G>C (rs17655) polymorphisms genotypes distinguished by PCR-RFLP. The product sizes after PCR amplification are as follows: (**a**) 152 bp. (**b**) 281 bp. (**c**) 324 bp. (**d**) 137 bp. (**e**) 129 bp. (**f**) 271 bp.

Peripheral venous blood samples were collected from each participant in the study (cases and controls) in EDTA (ethylene diamine tetra-acetic acid) tubes. Blood samples were used for genomic DNA extraction performed with the Quick-gDNA MiniPrep kits (Zymo Research, Irvine, CA, USA) and PureLink Genomic DNA Mini kits (Invitrogen, Carlsbad, CA, USA). The polymerase chain reaction–restriction fragment length polymorphism (PCR-RFLP) method was used in establishing the genotypes of *XPC* 1496C>T, *XPC* 2920A>C, *XPD* 2251A>C, *XPF*-673C>T, *XPF* 11985A>G, and *XPG* 3507G>C, as previously described [13,27,37,46,47,48]. After the PCR reaction, digestion was performed with specific restriction enzymes (Thermo Fisher Scientific, Waltham, MA, USA), followed by agarose gel electrophoresis (2%) (Table 3). The genotypes distinguished by PCR-RFLP are presented in Figure 1.

*JAK2* V617F and *CALR* mutations were performed as presented in previous papers [7,49,50]. *CALR* mutations were analyzed only in subjects negative for the *JAK2* V617F mutation; however, there were a few cases in which the mutant clone was in a small percentage, and testing was also performed for *CALR* mutations.

### 2.5. Statistical Methods

Numerical, continuous, and quantitative variables were described using mean ± standard deviation (SD) (minimum-maximum). Qualitative and categorical (nominal/ordinal) variables were described as absolute and relative frequencies (%) and were evaluated by Fisher’s exact test (two-sided) and the chi-square test to determine statistically significant differences between the two groups.

The normality of data distributions for genotype categories was analyzed by the One-Sample Kolmogorov–Smirnov test with Lilliefors Significance Correction. The statistical significance threshold was considered below 0.05 (*p*-value < 0.05). The odds ratios (ORs) and 95% confidence intervals (CIs) were used to evaluate the risk determined by the variant alleles. The univariate logistic regression model was used to analyze the predictive quality of the independent variables in the study. For the independent variables in the logistic regression model, the statistical significance threshold was considered below 0.05 (*p*-value < 0.05), with 95% confidence intervals for Exp (B) statistics. Statistical analysis was performed with SPSS 23.0 (licensed) software.

## 3. Results

### 3.1. Demographic Characteristics

Following the review of medical records, data were extracted regarding demographic characteristics, laboratory parameters, driver mutation status, clinical variables such as palpable splenomegaly, the presence of arterial and venous thrombosis, and leukemic progression (Table 4). The 393 patients with MPN included in the study were divided as follows: 153 with PV, 201 with ET, and 39 with PMF.

### 3.2. Distribution of Investigated XPC, XPD, XPF, and XPG SNPs in MPN Patients and Controls

Both the cases and the controls included in the study were successfully genotyped by PCR-RFLP. The genotype and allele frequencies of *XPC* 1496C>T, *XPC* 2920A>C, *XPD* 2251A>C, *XPF*-673C>T, *XPF* 11985A>G, and *XPG* 3507G>C and their association with the risk of developing MPN are shown in Table 5. There were no differences in the frequencies of the genotypes or the alleles of the *XPC* 1496C>T SNP between the control group and the MPN group (*p* = 0.91 for CT, *p* = 0.88 for TT, and *p* = 0.9 for T allele).

The clinical characteristics of MPN patients according to *XPC*, *XPD*, *XPF,* and *XPG* SNPs are presented in Table 4. For the *XPC* 1496C>T SNP, there was an association between the variant genotypes (CC + CT) and hematocrit (Htc > 48% in women (*p* = 0.048; OR = 0.49; 95% CI = 0.24−1). We found no associations between the *XPC* 1496C>T SNP and the clinical and hematological characteristics of the MPN patients (*p* > 0.05) (Table 6).

We did not observe a difference in the distribution of alleles or genotypes following the genotyping of the *XPC* 2920A>C polymorphism (*p* = 0.93 for C allele, *p* = 0.43 for AC, and *p* = 0.98 for CC). There was an association between aspirin use (*p* = 0.02; OR = 0.57; 95% CI = 0.36–0.9), hemoglobin value in women over 16 g/dL (*p* = 0.007; OR = 4.7; 95% CI = 1.38–16.04), hematocrit > 48% in women (*p* = 0.009; OR = 1.21; 95% CI = 1.08–1.36), the presence of non-myeloid neoplasms (*p* = 0.01; OR = 0.38; 95% CI = 0.18–0.84), and *XPC* 2920A>C SNP (Table 6). No associations were found between this polymorphism, gender, leukocytes, the presence of constitutional symptoms, and other characteristics (Table 6) (*p* > 0.05).

The heterozygous AC genotype (*XPD* 2251A>C SNP) presented an increased risk of developing MPN compared to controls (OR = 1.88; 95% CI = 1.35–2.61; *p* < 0.001). Also, variant genotypes (heterozygous plus homozygous) were associated with an increased risk of MPN (OR = 1.54; 95% CI = 1.15–2.08; *p* = 0.004). No difference was observed in the allele frequencies of *XPD* 2251A>C SNP between the two groups (*p* = 0.22).

A significant difference was observed in the allele frequency (OR = 0.71; 95% CI = 0.57–0.788; *p* = 0.002) between the two groups (*XPF*-673C>T SNP). None of the patients’ features (Table 6) were associated with the *XPF*-673C>T SNP. Variant genotypes were associated with a decreased risk of PV, ET, and PMF (heterozygous CT−OR = 0.5; 95% CI = 0.35–0.7; *p* < 0.001; CT + TT−OR = 0.56; 95% CI = 0.42–0.76; *p* < 0.001).

The heterozygous, homozygous variants and the combination of the two (AG, GG, and AG + GG) were associated with a decreased risk of MPN (OR = 0.3; 95% CI = 0.21–0.43; *p* < 0.001, OR = 0.19; 95% CI = 0.11–0.33; *p* < 0.00, and OR = 0.26; 95% CI = 0.19–0.37; *p* < 0.001) (*XPF* 11985A>G SNP). The variant allele of the *XPF* 11985A>G SNP may play a protective role against developing MPN (OR = 0.3; 95% CI = 0.23–0.39; *p* < 0.001).

No difference was observed in the frequencies of the genotypes of the *XPG* 3507G>C SNP between the MPN subjects and the controls (*p* = 0.47 for CC, and *p* = 0.94 for GC). The variant C allele was 22.6% in the control group and 21.62% in the patients’ group, and there was not a significant difference (*p* = 0.66). Leukocyte value ≥ 11 × 10^9^/L (*p* = 0.008) and bleeding history (*p* = 0.003; OR = 4.46; 95% CI = 1.46–8.23) were associated with variant genotypes of the *XPG* 3507G>C SNP (Table 6).

### 3.3. Possible Predictors for Patients Outcome

Considering that somatic mutations (*JAK2*, *CALR*) that occur in the neoplastic clone may maintain a chronic inflammatory state, prothrombotic status and constitutional symptoms have an increased susceptibility to secondary cancers and autoimmune disorders [51]; previous thrombotic events, age, leukocytosis, and the presence of *JAK2V617F* are predictive of MPN-associated thrombotic complications [52]. Also considering the fact that an increased rate of thrombosis is brought on by conventional cardiovascular risk factors [53], we analyzed the possible predictors for the outcome of the investigated MPN cases. The results of the logistic regression regarding the relationship between possible predictors and patients’ outcomes are presented in Table 7 and Table 8.

The results of the logistic regression presented in Table 7 show that the following variables—*XPD* 2251A>C (*p* = 0.004), *XPF-*673C>T (*p* < 0.001), and *XPF* 11985A>G (*p* < 0.001)—had a dependency relationship statistically significant to the MPN patients’ outcome. The other variables were not predictors for MPN patients’ outcome.

Table 8 presents possible predictors for the subgroups (PV, ET, and PMF). In the group of patients with PV, only hemoglobin value > 16.5 g/dL *p* < 0.001), male gender (*p* < 0.001), smoking (*p* = 0.035), and positive *CALR* mutation (*p* < 0.001) were predictors. In the group of patients with ET, male gender (*p* < 0.001), hemoglobin value > 16.5 g/dL (*p* < 0.001, positive *CALR* mutation (*p* < 0.001, smoking (*p* = 0.023), palpable splenomegaly (*p* = 0.001), and platelets > 450 × 10^9^/L (*p* < 0.001) were predictors. Platelet value > 450 × 10^9^/L (*p* < 0.001) was a predictor among patients with PMF.

## 4. Discussion

To investigate the association between the polymorphisms of the genes involved in the NER system with the appearance of MPN, we conducted this case–control study in a Romanian population.

Allele frequencies in the patient group were similar to those reported at the European level (Table 2). No association was observed between the variant genotypes of *XPC* 1496C>T and MPN risk in the studied population. Similar to our findings, Thakkar et al. found no association between variant genotypes of *XPC* 1496C>T SNP and the risk of developing Hodgkin lymphoma in a population from South India [54]. Also, no association was observed between *XPC* 1496C>T polymorphism and the risk of myelodysplastic syndrome [15] and with the risk of AML conversion from ET and PV [26]. Different results were reported by Monroy et al., who reported that the heterozygous CT genotype had been associated with an increased risk of Hodgkin lymphoma (OR = 1.77; 95% CI =1.17–2.68) [25].

In this study, we noticed that *XPC* 2920A>C is not a risk factor for developing MPN. Similar results were obtained by Kim et al. in patients diagnosed with non-Hodgkin’s lymphoma [55], and in another study with cases with Hodgkin lymphoma subjects (*p* = 0.122) [54]. It was suggested that variant genotypes of *XPC* 2920A>C may have a protective role in non-smokers against lymphoma (*p* = 0.04) [56]. In a US study of a cohort of 200 subjects, no association was found between *XPC* 2920A>C SNP and the risk of developing Hodgkin’s disease. Despite this, the association between *XRCC1* Arg/Gln and *XPC* Lys/Lys was found to decrease the risk of developing Hodgkin’s disease (OR = 2.14; 95% CI = 1.09−4.23) [57]. Also, in a study performed on the Romanian population, no association was reported between the variant genotypes of *XPC* 2920A>C and the risk of developing AML [29]. A strong association between *XPC* 2920A>C and *XPC* 1496C>T SNPs and response to imatinib treatment has been reported for 92 Caucasian patients with chronic myeloid leukemia (CML) [58]. Different results were presented by Douzi et al. in a study in which homozygous variant genotypes of *XPC* 2920A>C were associated with a high risk of developing leukemia (OR = 2.484; 95% CI = 1.35–4.56) [27].

Variant genotypes (AC + CC) of *XPD* 2251A>C were associated with an increased risk of developing MPN (OR = 1.55; 95% CI = 1.145–2.08; *p* = 0.004). Data similar to ours were obtained in a study on a Romanian population in which the variant genotypes of *XPD* were associated with an increased risk of developing AML (OR = 2.55; 95% CI = 1.53–4.25) [29]. Following a meta-analysis performed by Liu et al. on 3753 subjects, the results showed the possibility that *XPD* 2251A>C may be a risk factor for AML, especially for Caucasian patients with acute leukemia (OR = 1.23; 95% CI = 1.03–1.46) [59]. Another study of 156 Romanian patients with CML showed an association between variant genotypes and the risk of developing CML (OR = 1.72; 95% CI = 1.10–2.69) [33].

The study conducted on a Spanish population showed that the homozygous variant genotypes of *XPD* 2251A>C are associated with an increased risk of transformation to AML [26]. Exposure to cytoreductive treatments, patient age, and leukocytosis at diagnosis are considered risk factors for progression to acute leukemia in patients with PV and ET [60]. In contrast, the research conducted by Poletto et al. which included 456 Italian MFP patients did not report any association between *XPD* 2251A>C and the risk of leukemic transformation [34].

The data presented by Chen et al. following the case–control study in Connecticut revealed that the women with a BMI (body mass index) > 25 who carried the AA genotype of *XPD* 2251A>C had a significantly lower risk of developing NHL (OR = 2; 95% CI = 1.4–3) [61]. *XPD* 2251A>C was associated with lower overall survival for diffuse large B-cell lymphoma (DLBCL) in a study of a US population [62].

Different results from ours were obtained in a study with Asian patients, 694 with non-Hodgkin’s lymphoma (NHL), 378 with DLBCL, and 140 with T-cell lymphoma. No association was obtained between *XPD* 2251A>C and T-cell lymphoma, DLBCL, and NHL [36]. In a meta-analysis of 3095 patients with NHL and 3306 controls conducted on a Caucasian, Asian, and mixed ethnicities population, no significant association between *XPD* 2251A>C polymorphism and the risk of Hodgkin’s lymphoma was brought to light [30].

Dhangar et al. conducted a study of 87 Indian patients diagnosed with CML, and no association between treatment response and *XPD* 2251A>C was reported [32]. An analysis of leukemia subtypes in the study by Douzi et al. on a Tunisian population showed that the variant allele of *XPD* 2251A>C was a protective factor and was associated with a lower risk of developing CML [27]. Similar results were reported on Egyptian controls and patients with AML [36]. Moreover, no association between variant homozygous genotypes of *XPD* 2251A>C with various hematological malignancies such as acute lymphoblastic leukemia (ALL), AML, NHL, and Hodgkin’s Lymphoma (HL) was found in a Turkish population [63].

In addition, we observed that blood emissions (*p* = 0.03; OR = 2.2; 95% CI = 1.1–4.6) and aspirin use (*p* = 0.005; OR = 1.86; 95% CI = 1.2–2.9) were found to be associated with *XPD* 2251A>C polymorphism, and history of thrombosis (*p* = 0.044; OR = 0.66; 95% CI = 0.41–0.99) was negatively associated with this SNP (Table 6). The other characteristics were not associated with this SNP (Table 6).

In the present study, the variant genotypes of the *XPF*-673C>T polymorphism were associated with a low risk of developing MPN (OR = 0.56; 95% CI = 0.42–0.76). Similar results were reported previously in AML (OR = 0.57; 95% CI = 0.34–0.98 [29]. Also, the TT genotype of *XPF*-673C>T was associated with a decreased esophageal squamous cell carcinoma risk in the Chinese population among the non-smoker group, but not among the smoker group [37]. An old study, conducted by Shao, showed that variant genotypes of *XPF*-673C>T SNP significantly increased the risk of lung cancer in non-smokers, but not in smoker patients [64]. In contrast to the results presented by Shao, Yu et al. did not bring to light associations between *XPF*-673C>T and smoking [65].

In our research, both homozygous and heterozygous *XPF* 11985A>G variant genotypes appear to be associated with a low risk of developing MPN (OR = 0.19; 95% CI = 0.11–0.33 and OR = 0.3; 95% CI = 0.21–0.43). Similar results were obtained for the heterozygous variant genotype in AML patients (OR = 0.22; 95% CI = 0.09–0.51) [29]. For *XPF* 11985A>G polymorphism, according to the results presented by Liu et al., no significant differences were found between patients with esophageal squamous cell carcinoma and controls (*p* = 0.2 for AG genotype; *p* = 0.36 for GG genotype) [37]. These results are in contradiction with our findings. One explanation may be the different ethnicity (Caucasians versus Asians) and investigated disorders (MPN versus esophageal squamous cell carcinoma).

Moreover, our data showed that exposure to an HU or HU in combination with other agents has been associated with variant *XPF* 11985A>G genotypes (*p* = 0.003). Leukemic transformations were found predominantly in patients with PMF (*n* = 9; 23.7%), while in patients with ET and PV, there were lower percentages: 7.46% and 5.33%, respectively. Leukemic transformations have been associated with variant genotypes of the *XPF* 11985A>G SNP (*p* = 0.04) (Table 6).

In the present study, variant genotypes of *XPG* 3507G>C were not associated with the risk of developing MPN. Our findings are consistent with those reported by ElMahgoub following a study of 50 Egyptian patients with acute leukemia [13]. As a similarity, Ruiz-Cosano et al., following a study of 213 cases and 214 controls, reported that *XPG* 3507G>C polymorphism was not associated with the risk of lymphoma (OR = 1.1; 95% CI = 0.8–1.7) [66]. Also, comparable results were obtained in a study with patients diagnosed with polycythemia vera and essential thrombocythemia in which this SNP was not associated with the risk of leukemic transformation [26]. Al Sayed Ahmed et al. showed through their study a significant difference in the distribution of allele frequency between the control group and the group of patients with classic Hodgkin’s lymphoma [67].

The association of variant homozygous genotypes of *XPC* 939 Gln/Gln and *XPG* 1104 His/His polymorphisms led to significant interaction with the risk of leukemia, especially in the case of CML (OR = 22.52; 95% CI = 5.38–94.25 [27]. The results are similar to those obtained by El-Zein et al. in a study that included 200 subjects diagnosed with Hodgkin’s lymphoma [57]. The heterozygous genotypes of *XPG* 3507G>C were associated with a risk of developing AML in a study performed on a Romanian population (OR = 2.36; 95% CI = 1.33–4.22) [29]. Different results were obtained in a study conducted by Bahceci et al., which found that variant genotypes of this SNP have a protective role for lymphoma (OR = 0.47; 95% CI = 0.26–0.84) [56]. Contrary to the results of different studies performed on different disorders, the present research showed no association between *XPC* 2920A>C and the risk of developing MPN.

The results of the logistic regression (Table 7) revealed that three variables, namely *XPD* 2251A>C (*p* = 0.004), *XPF*-673C>T (*p* < 0.001), and *XPF* 11985A>G (*p* < 0.001), had a dependency relationship statistically significant to the MPN patients’ outcome. Also, male gender (*p* < 0.001), positive *CALR* mutation (*p* < 0.001), smoking (*p* = 0.023), hemoglobin value > 16.5 g/dL, platelet value > 450 × 10^9^/L (*p* < 0.001), and palpable splenomegaly (*p* = 0.001) were predictors in the group of patients with ET, while in the group of patients with PV, only male gender (*p* < 0.001), positive *CALR* mutation (*p* < 0.001), smoking (*p* = 0.035), and hemoglobin value > 16.5 g/dL were predictors. Platelet value > 450 × 10^9^/L (*p* < 0.001) was a predictor among patients with PMF (Table 8).

According to the literature, between 96% and 99% of PV patients have a *JAK2* mutation, and therefore *CALR* mutations should be absent or very rare. It has been shown that in some cases, *JAK2-V617F* and *CALR* mutations can coexist [68]. In our study, we describe a patient with PV who was *JAK2-V617F*-negative but had a *CALR* mutation (Table 4).

Although different treatment options for MPN exist, including targeted therapy (Ruxolitinib or Jafaki, a drug that targets *JAK2*), chemotherapy, and immunotherapy, resistance to treatment inevitably occurs. The identification of risk alleles of genes involved in NER may lead to the development of novel target therapies that may improve the outcome of the patients. For example, Poly (ADP-ribose) polymerase (PARP) inhibitors target DNA repair damage and are a promising treatment in lung cancer [69,70].

Mutations of the genes involved in NER were recently investigated by whole exome sequencing and were reported to be associated with different types of cancers and to have a potential impact on clinical outcomes [70]. It was reported that NER inhibition confers increased sensitivity to cisplatin (alkylating agents) and may be an additional target that could be used in combination therapies [71].

Studies with similar designs showed different results; possible causes could be etiologies and genetic backgrounds, as well as ethnic diversity. A limitation of our study is the relatively low number of MPN patients, especially in the PMF subgroup. Another weak point is the fact that the patients come from only one region of Romania.

To our knowledge, this is the first study that investigated the following six SNPs (*XPC* 1496C>T, *XPC* 2920A>C, *XPD* 2251A>C, *XPF-*673C>T, *XPF* 11985A>G, and *XPG* 3507G>C) involved in the etiology of MPN patients and also analyzed the relation between investigated polymorphisms and *JAK2*-V617F or *CALR* driver mutations.

## 5. Conclusions

Based on the data obtained in the current study, we consider that *XPD* 2251A>C may influence MPN and that *XPF-*673C>T and *XPF* 11985A>G single nucleotide polymorphisms (SNPs) had a protective role for MPN, while *XPC* 1496C>T, *XPC* 2920A>C, and *XPG* 3507G>C polymorphisms do not represent risk factors in MPN development.

According to our findings, the variant *XPD* 2251A>C, *XPF*-673C>T, and *XPF* 11985A>G genotypes represent independent predictors for MPN. Also, *CALR* gene mutation, male gender, platelet value, palpable splenomegaly, smoking, and hemoglobin value represent independent predictors for patients with ET. Male gender, positive *CALR* mutation, smoking, and hemoglobin value were predictors for patients with PV. Platelet value was a predictor among patients with PMF.

Further research with a large cohort of patients belonging to all geographical regions of Romania should clarify the conclusions regarding the link between the six gene polymorphisms of the NER system and MPN.

## Figures and Tables

**Table 1:** Distribution of demographic data of MPN patients and controls.

Variable	MPN Patients (*n* = 393)	Controls (*n* = 323)	*p*-Value
Gender			
Male gender [*n* (%)]	188 (47.8)	155 (48)	0.96
Female gender [*n* (%)]	205 (52.2)	168 (52)	
Age			
Age at diagnosis, years; median	60 (17–85)	56.15 (25–94)	
≥60 [*n* (%)]	199 (50.6)	194 (49.4)	0.11
<60 [*n* (%)]	194 (49.4)	179 (55.4)	

*n*—number of patients; *p*-values obtained using ANOVA test; *p*-value < 0.05 was considered significant.

**Table 2:** Data about ID, allele frequencies, clinical significance of the investigated SNPs.

Gene Polymorphism	rs ID	MAF	Risk Allele Frequency ALL		Risk Allele Frequency in Europe		Most Severe Consequence	Clinical Significance
			WT-Allele	Variant	WT-Allele	Variant		
*XPC* 1496C>T	rs2228000	0.48	G—0.77	A—0.23	G—0.74	A—0.26	Missense variant	Benign
*XPC* 2920A>C	rs2228001	0.49	G—0.32	T—0.66	G—0.40	T—0.60	Missense variant	Benign, likely benign
*XPD* 2251A>C	rs13181	0.45	T—0.76	G—0.24	T—0.64	G—0.36	Stop gained	Benign, likely benign
*XPF-*673C>T	rs3136038	0.49	C—0.66	T—0.34	C—0.66	T—0.34	TF binding site	-
*XPF* 11985A>G	rs254942	0.25	G—0.05	A—0.95	G—0.02	A—0.98	Splice region variant	Benign
*XPG* 3507G>C	rs17655	0.5	G—0.64	C—0.36	G—0.75	C—0.25	Missense variant	Benign

WT—wild type, MAF—minor allele frequency.

**Table 3:** PCR-RFLP description (restriction enzyme, genotypes, length of the PCR products after digestion, and primers used).

Gene Polymorphism	Restriction Enzyme Used	Base Pair Change	Genotype	Length (bp)	Primers Sequences
*XPC* 1496C>T (*XPC* Ala499Val, rs2228000)	Cfr42I (SacII)	C→T	CC	131, 21	Fw: TAA GGA CCC AAG CTT GCC CG Rev: CCC ACT TTT CCT CCT GCT CAC AG
			CT	152, 131, 21	
			TT	152	
*XPC* 2920A>C (*XPC* Lys939Gln, rs2228001)	Pvu II	A→C	AA	281	Fw: GAT GCA GGA GGT GGA CTC TCT Rev: GTA GTG GGG CAG CAG CAA CT
			AC	281, 150, 131	
			CC	150, 131	
*XPD* 2251A>C (*XPD* Lys751Gln, rs13181)	Pst I	A→C	AA	224, 100	Fw: TC CTG TCC CTA CTG GCC ATT C Rev: GT GGA CGT GAC AGT GAG AAA T
			AC	224, 158, 100, 66	
			CC	158, 100, 66	
*XPF-*673C>T (rs3136038)	EcoRI	C→T	CC	114, 23	Fw: GGG AGG CAA ACA GAG GTC TGA ATT Rev: TGC GAT TAC TCC CCA TCC TTC TT
			CT	137, 114, 23	
			TT	137	
*XPF* 11985A>G (rs254942)	RsaI	A→G	AA	129	Fw: GGA GTC AAG AAA CAG CCA ACC TAG TA Rev: AGG AAG ACA GGA TGA CAG CCA G
			AG	129, 104, 25	
			GG	104, 25	
*XPG* 3597G>C (*XPG* Asp1104His, rs17655)	NlaIII (Hin1 II)	G→C	GG	271	Fw: GAC CTG CCT CTC AGA ATC ATC Rev: CCT CGC ACG TCT TAG TTT CC
			GC	271, 227, 44	
			CC	227, 44	

PCR-RFLP—Polymerase chain reaction–restriction fragment length polymorphism; FW—Forward, Rev—Reverse.

**Table 4:** Demographic characteristics, laboratory parameters, driver mutation status, clinical variables of MPN patients.

Characteristics	Patients with PV (*n* = 153)	Patients with ET (*n* = 201)	Patients with PMF (*n* = 39)	All Patients (*n* = 393)
Age at diagnosis, years, median (range)	59 (17–80)	60 (18–85)	59 (34–76)	60 (17–85)
< 30 [*n* (%)]	10 (6.54)	7 (3.49)	-	17 (4.32)
30–49 [*n* (%)]	29 (18.95)	48 (23.89)	7 (17.95)	84 (21.38)
50–69 [*n* (%)]	85 (55.55)	94 (46.77)	27 (69.23)	206 (52.42)
≥ 70 [*n* (%)]	29 (18.96)	52 (25.87)	5 (12.82)	86 (21.88)
Gender				
Male [*n* (%)]	94 (61.43)	75 (37.31)	19 (48.71)	188 (47.83)
Female [*n* (%)]	59 (38.57)	126 (62.69)	20 (51.29)	205 (52.17)
Blood counts				
Hemoglobin (g/dL), median (range)	17.2 (7.7–22.7)	13.2 (4.8–20)	10 (5.9–14.5)	14.4 (4.8–22.7)
Hemoglobin < 10 g/dL [*n* (%)]	3 (1.96)	35 (17.41)	19 (48.72)	57 (14.50)
Hemoglobin 10–16.5 g/dL [*n* (%)]	54 (35.30)	160 (79.61)	20 (51.28)	234 (59.53)
Hemoglobin > 16.5 g/dL [*n* (%)]	96 (62.74)	6 (2.98)	-	106 (26.97)
Hematocrit value, median (range)	50.91 (24.3–73.4)	39.5 (6.29–55.6)	31.7 (18.9–46.3)	43.4 (6.29–73.4)
Hematocrit > 49 [*n* (%)]	89 (58.17)	13 (6.47)	0	102 (25.95)
Hematocrit ≤ 49 [*n* (%)]	64 (41.83)	188 (93.53)	39 (100)	291 (74.05)
Red blood cells median (range)	5.74 (2.7–9.3)	4.37 (1.86–9)	3.41 (2.22–5.63)	4.71 (1.86–9.3)
Platelets (×10^9^/L), median (range)	282 (77–1619)	720 (34–3160)	260 (4–1167)	543 (4–3160)
Platelets < 100 × 10^9^/L [*n* (%)]	3 (1.97)	1 (0.49)	8 (20.51)	13 (3.3)
Platelets 100–450 × 10^9^/L [*n* (%)]	113 (73.85)	16 (7.96)	25 (64.11)	154 (39.19)
Platelets > 450 × 10^9^/L [*n* (%)]	37 (24.18)	184 (91.55)	6 (15.38)	226 (57.51)
Leukocytes (×10^9^/L), median (range)	9.88 (3.44–182.3)	9.51 (3.59–113.83)	9.5 (0.6–82.30)	9.67 (0.6–182.3)
Leukocytes < 11 × 10^9^/L [*n* (%)]	88 (57.51)	125 (62.19)	22 (56.41)	235 (59.8)
Leukocytes ≥ 11 × 10^9^/L [*n* (%)]	65 (42.48)	76 (37.81)	17 (43.58)	158 (40.20)
Leukocytes 11–15 × 10^9^/L [*n* (%)]	30 (19.6)	44 (21.89)	6 (15.38)	80 (22.36)
Leukocytes ≥ 15 × 10^9^/L [*n* (%)]	35 (22.87)	32 (15.92)	11 (28.20)	78 (19.84)
Leukocytes 15–25 × 10^9^/L [*n* (%)]	24 (15.7)	19 (9.45)	5 (12.82)	48 (12.21)
Leukocytes ≥ 25 × 10^9^/L [*n* (%)]	11 (7.19)	13 (6.47)	6 (15.39)	30 (7.63)
LDH median U/L (range)	284 (102–2015)	308 (113–2197)	379 (130–3098)	307 (102–3098)
Driver mutational status				
*JAK2* mutation [*n* (%)]	69 (45.09)	88 (43.78)	17 (43.59)	174 (44.27)
*CALR* mutation [*n* (%)]	1 (0.65)	37 (18.4)	8 (20.51)	46 (11.7)
2x-negative [*n* (%)]	68 (44.44)	72 (35.8)	13 (33.33)	153 (38.93)
Constitutional symptoms [*n* (%)]	73 (47.71)	99 (49.25)	25 (64.1)	197 (50.12)
Palpable splenomegaly [*n* (%)]	66 (43.13)	66 (32.83)	29 (74.35)	161 (40.96)
History of any thrombosis [*n* (%)]	47 (30.71)	61 (30.34)	10 (25.64)	118 (30.02)
History of venous thrombosis [*n* (%)]	22 (14.37)	22 (10.94)	8 (20.51)	52 (13.23)
History of arterial thrombosis [*n* (%)]	32 (20.91)	43 (21.39)	5 (12.82)	80 (20.35)
History of bleeding [*n* (%)]	6 (3.92)	14 (6.96)	5 (12.82)	25 (6.35)
Leukemic transformations [*n* (%)]	8 (5.22)	15 (7.46)	9 (23.07)	32 (8.11)

*n*—number of patients.

**Table 5:** Genotypes distribution of *XPC*, *XPD*, *XPF*, and *XPG* polymorphisms in MPN patients and controls.

	MPN Patients *n*-393 (%)	Controls *n*-323 (%)	Crude OR (95% CI)	*p*-Value
*XPC* 1496C>T (rs2228000, Ala499Val)				
CC	180 (45.8)	148 (45.8)	Ref.	Ref.
CT	134 (34.1)	108 (33.4)	1.02 (0.731–1.425)	0.907
TT	79 (20.1)	67 (20.7)	0.969 (0.655–1.434)	0.877
CT + TT	213 (54.2)	175 (54.1)	1.001 (0.745–1.345)	0.996
C allele	494 (62.84)	404 (62.53)	Ref.	Ref.
T allele	292 (37.15)	242 (37.46)	0.986 (0.795–1.224)	0.903
*XPC* 2920A>C (rs2228000, XPC Lys939Gln)				
AA	104 (26.5)	79 (24.5)	Ref.	Ref.
AC	204 (51.9)	179 (55.4)	0.866 (0.607–1.234)	0.425
CC	85 (21.6)	65 (20.1)	0.993 (0.642–1.536)	0.976
AC + CC	289 (73.5)	244 (75.5)	0.9 (0.641–1.262)	0.541
A allele	412 (52.41)	337 (52.17)	Ref.	Ref.
C allele	374 (47.58)	309 (47.83)	0.99 (0.804–1.219)	0.925
*XPD* 2251A>C *(rs*13181, *XPD* Lys751Gln)				
AA	147 (37.4)	155 (48)	Ref.	Ref.
AC	185 (47.1)	104 (32.2)	1.876 (1.349–2.608)	*<0.001*
CC	61 (15.5)	64 (19.8)	1.005 (0.662–1.525)	0.981
AC + CC	246 (62.6)	168 (52)	1.544 (1.145–2.082)	*0.004*
A allele	479 (60.94)	414 (64.08)	Ref.	Ref.
C allele	307 (39.05)	232 (35.91)	1.144 (0.922–1.418)	0.222
*XPF-*673C>T (rs3136038)				
CC	212 (53.9)	128 (39.6)	Ref.	Ref.
CT	106 (27)	129 (39.9)	0.496 (0.354–0.696)	*<0.001*
TT	75 (19.1)	66 (20.4)	0.686 (0.461–1.020)	0.062
CT + TT	181 (46.1)	195 (60.3)	0.56 (0.416–0.755)	*<0.001*
C allele	530 (67.43)	385 (59.59)	Ref.	Ref.
T allele	256 (32.56)	261 (40.4)	0.712 (0.573–0.884)	*0.002*
*XPF* 11985A>G (rs254942)				
AA	313 (79.6)	164 (50.8)	Ref.	Ref.
AG	62 (15.8)	109 (33.7)	0.298 (0.207–0.429)	*<0.001*
GG	18 (4.6)	50 (15.5)	0.189 (0.107–0.334)	*<0.001*
AG + GG	80 (20.4)	159 (49.2)	0.264 (0.190–0.366)	*<0.001*
A allele	688 (87.53)	437 (67.64)	Ref.	Ref.
G allele	98 (12.46)	209 (32.35)	0.297 (0.227–0.389)	*<0.001*
*XPG* 3507G>C (rs17655, XPG Asp1104His)				
GG	236 (60.1)	191 (59.1)	Ref.	Ref.
GC	144 (36.6)	118 (36.5)	0.988 (0.725–1.346)	0.937
CC	13 (3.3)	14 (4.3)	0.752 (0.345–1.637)	0.471
GC + CC	157 (39.9)	132 (40.8)	0.963 (0.713–1.299)	0.803
G allele	616 (78.37)	500 (77.4)	Ref.	Ref.
C allele	170 (21.62)	146 (22.6)	0.9452 (0.7356–1.215)	0.658

Ref.—reference; *n*—number of patients; *p*-values obtained from chi-square test, *p*-value < 0.05 was considered significant and is indicated in italics.

**Table 6:** Patient features at diagnosis according to the *XPC*, *XPD*, XPF, and *XPG* genotypes.

Characteristics	All Patients [*n* (%)]	*XPC* 1496C>T			*XPC* 2920A>C			*XPD* 2251A>C			*XPF-*673C>T			*XPF* 11985A>G			*XPG* 3507G>C		
		CC	Variant TT + CT	*p-*Value	AA	Variant CC + AC	*p-*Value	AA	Variant TT + AC	*p-*Value	CC	Variant TT + CT	*p-*Value	AA	Variant GG + AG	*p-*Value	GG	Variant CC + GC	*p-*Value
Mutations																			
*JAK2+*	174 (44.27)	86	88	0.2	42	132	0.35	60	114	0.29	96	78	0.66	140	34	0.72	105	69	0.92
*JAK2−*	219 (55.72)	94	125		62	157		87	132		116	103		173	46		131	88	
*CALR+*	46 (11.7)	21	25	0.98	12	34	0.95	18	28	0.8	28	18	0.32	40	6	0.19	30	16	0.45
*CALR−*	347 (88.29)	159	188		92	255		129	218		184	163		273	74		206	141	
Subtype																			
PV	153 (38.93)	73	80	0.71	38	115	0.84	58	95	0.85	86	67	0.76	121	32	0.47	96	57	0.62
ET	201 (51.14)	88	113		55	146		73	128		106	95		158	43		116	85	
MPF	39 (9.92)	19	20		11	28		16	23		20	19		34	5		24	15	
Gender																			
Male	188 (47.8)	83	105	0.53	49	139	0.86	69	119	0.78	104	84	0.6	144	44	0.15	119	69	0.21
Female	205 (52.2)	97	108		55	150		78	127		108	97		169	36		117	88	
Constitutional symptoms																			
Present	197 (50.12)	93	104	0.58	58	139	0.18	74	123	0.95	111	86	0.34	157	40	0.98	114	83	0.38
Absent	196 (49.87)	87	109		46	150		73	123		101	95		156	40		122	74	
Palpable splenomegaly																			
Present	161 (40.96)	73	88	0.88	41	120	0.71	54	107	0.19	117	115	0.09	134	27	0.14	104	57	0.13
Absent	232 (59.03)	107	125		63	169		93	139		95	66		179	53		132	100	
Exposure to cytoreductive agents																			
Agents alone or in combination	160 (40.71)	80	80	0.17	46	114	0.4	56	104	0.41	91	69	0.33	139	21	*0.003*	100	60	0.41
No exposure	233 (59.28)	100	133		58	175		91	142		121	112		174	59		136	97	
Blood emissions																			
	Yes	24	20	0.22	11	33	0.82	10	34	*0.03*	25	19	0.69	37	7	0.44	31	13	0.14
No	349 (88.8)	156	193		93	256		137	212		187	162		276	73		205	144	
Aspirine																			
Yes	150 (38.16)	67	83	0.72	50	100	*0.02*	43	107	*0.005*	77	73	0.42	117	33	0.53	90	60	0.99
No	243 (61.83)	113	130		54	189		104	139		135	108		196	47		146	97	
Interferon Alfa																			
Yes	5 (1.27)	3	2	0.52	3	2	0.09	3	2	0.29	3	2	0.79	5	0	0.26	2	3	0.36
No	388 (98.78)	177	211		101	287		144	244		209	179		308	80		234	154	
Hemoglobin in Males																			
Hemoglobin > 16.5 g/dL	75 (19.08)	29	46	0.22	24	51	0.13	31	44	0.28	39	36	0.46	58	86	0.85	46	29	0.65
Hemoglobin ≤ 16.5 g/dL	113 (28.75)	54	59		25	88		38	75		65	48		17	27		73	40	
Hemoglobin in Females																			
Hemoglobin > 16 g/dL	35 (8.91)	19	16	0.37	3	32	*0.007*	16	19	0.31	23	12	0.09	29	6	0.94	19	16	0.71
Hemoglobin ≤ 16 g/dL	170 (43.26)	78	92		52	118		62	108		85	85		140	30		98	72	
Hematocrit in Males																			
Hematocrit > 49%	118 (30.03)	26	41	0.26	23	44	*0.05*	28	39	0.34	34	33	0.31	51	90	0.98	38	29	0.13
Hematocrit ≤ 49%	67 (17.05)	56	62		25	93		41	77		69	49		16	28		80	38	
Hematocrit in Females																			
Hematocrit > 48%	39 (9.92)	24	15	*0.048*	4	35	*0.009*	17	22	0.43	23	16	0.38	33	6	0.69	20	19	0.42
Hematocrit ≤ 48%	166 (42.24)	73	93		51	115		61	105		85	81		136	30		97	69	
Platelets (×10^9^/L)																			
Platelets > 450 × 10^9^/L	227 (57.76)	104	122	0.92	56	170	0.38	86	140	0.76	120	106	0.7	181	45	0.8	128	98	0.11
Platelets ≤ 450 × 10^9^/L	166 (42.23)	76	91		48	119		61	106		92	75		132	35		108	59	
Leukocytes (×10^9^/L)																			
Leukocytes ≥ 11 × 10^9^/L	80 (20.35)	39	41	0.81	18	62	0.85	30	50	0.73	42	38	0.99	61	19	0.41	54	26	*0.008*
Leukocytes ≥ 25 × 10^9^/L	48 (12.21)	21	27		9	39		21	27		25	23		41	7		22	26	
Leukocytes ≥ 15 × 10^9^/L	30 (7.63)	13	17		7	23		11	19		16	14		25	5		13	17	
Leukemic transformations																			
Yes	32 (8.14)	12	20	0.33	10	22	0.52	15	17	0.23	16	16	0.64	21	11	*0.04*	21	11	0.5
No	361 (91.85)	168	193		94	267		132	229		196	165		292	69		215	146	
Nonmyeloid malignancies																			
Yes	28 (7.12)	12	16	0.75	13	15	*0.01*	8	20	0.32	16	12	0.73	19	9	0.19	18	10	0.64
No	365 (92.87)	168	197		91	274		139	226		196	169		294	71		218	147	
Smoking habits																			
Yes	118 (30.02)	58	60	0.38	31	87	0.955	44	74	0.98	72	46	0.07	101	17	0.06	70	48	0.85
No	275 (69.97)	122	153		73	202		103	172		140	135		212	63		166	109	
Alcohol habits																			
Regular	8 (2.03)	2	6	0.06	2	6	0.31	3	5	0.99	4	4	0.88	5	3	0.41	3	5	0.35
Social	33 (17.3)	39	29		13	55		25	43		35	33		56	12		39	29	
Never	144 (80.66)	139	178		89	228		119	198		173	144		252	65		194	123	
Exposure to noxes																			
Yes	44 (11.19)	19	25	0.71	10	255	0.55	18	26	0.61	26	18	0.47	37	7	0.44	22	22	0.15
No	349 (88.8)	161	188		94	34		129	220		186	163		276	73		214	135	
History of bleeding																			
Yes	25 (6.36)	12	13	0.82	7	18	0.86	7	18	0.32	9	16	0.06	22	3	0.28	8	17	*0.003*
No	368 (93.63)	168	200		97	271		140	228		203	165		291	77		228	140	
History of any thrombosis																			
Yes	118 (30.02)	54	64	0.99	31	87	0.96	53	65	*0.04*	61	57	0.56	94	24	1	66	52	0.28
No	275 (69.97)	126	149		73	202		94	181		151	124		219	56		170	105	
History of venous thrombosis																			
Yes	52 (13.23)	21	31	0.4	13	39	0.8	24	28	0.16	24	28	0.23	38	14	0.21	30	22	0.71
No	86 (86.76)	159	182		91	250		123	218		188	153		275	66		206	135	
History of arterial thrombosis																			
Yes	80 (20.35)	41	39	0.27	20	60	0.74	34	46	0.29	41	39	0.59	67	13	0.31	46	34	0.6
No	313 (79.64)	139	174		84	229		113	200		171	142		246	67		190	123	

*p*-values obtained from chi-square tests and *p*-values < 0.05 were considered significant and are indicated in italics.

**Table 7:** Results of the logistic regression regarding the relationship between possible predictors and patient outcome (MPN group).

Possible Predictors		MPN	
	*n* (%)	*p*-value	Crude OR (95% CI)
Age ≥ 60 years	199 (50.6)	0.11	1.28 (0.95–1.71)
Gender (male)	188 (47.8)	0.97	1.006 (0.75–1.35)
*XPC* Ala499Val (variant)	213 (54.2)	0.97	0.99 (0.74–1.34)
*XPC* Lys939Gln (variant)	289 (73.5)	0.54	1.11 (0.79–1.56)
*XPD* Lys751Gln (variant)	246 (62.6)	*0.004*	0.65 (0.48–0.87)
*XPF-*673C>T (variant)	181 (46.1)	*<0.001*	1.78 (1.32–2.41)
*XPF* 11985A>G (variant)	80 (20.4)	*<0.001*	3.79 (1.32–2.41)
*XPG* Asp1104His (variant)	157 (39.9)	0.8	1.039 (0.77–1.40)

Reference categories: Age < 60 years; gender = female; *XPC 1496C>T* variant—TT + CT; *XPC* 2920A>C variant—CC + CT; *XPD* 2251A>C variant—CC + AC; *XPF*-673C>T variant—TT + CT; *XPF* 11985A>G variant—GG + AG; *XPG* 3507G>C variant—CC + GA; *p*-value < 0.05 was considered significant and is indicated in italics.

**Table 8:** Results of the logistic regression regarding the relationship between possible predictors and patient outcome (PV, ET, PMF groups).

Possible Predictors	PV Patients with PV (%)	PV		Patients with ET (%)	ET		Patients with PMF (%)	PMF	
		*p*-Value	Crude OR (95% CI)		*p*-Value	Crude OR (95% CI)		*p*-Value	Crude OR (95% CI)
Age ≥60 years	72 (47.05)	0.22	1.29 (0.86–1.94)	109 (54.22)	0.15	0.75 (0.5–1.11)	19 (52.77)	0.8	1.09 (0.56–2.11)
Gender (male)	94 (61.43)	*<0.001*	4.42 (1.6–3.67)	*75 (37.31)*	*<0.001*	0.42 (0.28–0.62)	19 (48.71)	0.91	1.04 (0.54–2.02)
*JAK2* (positive)	69 (45.09)	0.88	0.97 (0.65–1.46)	88 (43.78)	0.84	1.04 (0.7–1.55)	174 (43.59)	0.93	1.03 (0.53–2)
*CALR* (positive)	1 (0.65)	*<0.001*	34.67 (4.75–254.37)	*37 (18.4)*	*<0.001*	0.22 (0.102–0.47)	8 (20.51)	0.08	0.47 (0.2–1.08)
Smoking habits	56 (36.6)	*0.035*	0.63 (0.40–0.98)	50 (24.87)	0.023	1.66 (1.07–2.56)	12 (30.76)	0.92	0.96 (0.47–1.97)
Alcohol habits	28 (18.3)	0.72	1.10 (0.66–1.85)	41 (20.40)	l0.87	0.87 (0.53–1.44)	7 (17.95)	0.82	1.11 (0.47–2.61)
Hemoglobin > 16.5 g/dL	96 (62.74)	*<0.001*	0.018 (0.008–0.041)	*6 (2.98)*	*<0.001*	32.5 (13.75–76.82)	-	-	-
Platelets > 450 × 10^9^/L	37 (24.18)	0.41	1.77 (0.46–6.85)	184 (91.54)	*<0.001*	0.021 (0.003–1.67)	6 (15.38)	*<0.001*	73.67 (17.32–31.58)
Leukocytes ≥ 11 × 10^9^/L	65 (42.48)	0.41	1.77 (0.46–6.85)	76 (37.81)	0.275	1.25 (0.84–1.88)	17 (43.58)	0.63	0.85 (0.43–1.65)
Exposure to cytoreductive agents	63 (41.17)	0.98	0.99 (0.66–1.5)	81 (40.29)	0.86	1.03 (0.69–1.55)	16 (41.02)	0.97	0.99 (0.5–1.93
Exposure to noxious substances	14 (9.15)	0.27	0.7 (0.37–1.32)	26 (12.93)	0.27	0.7 (0.37–1.32)	4 (10.25)	0.85	1.12 (0.38–3.3)
Palpable splenomegaly	66 (43.13)	0.57	0.89 (0.59–1.34)	66 (32.83)	*0.001*	2 (1.33–3.01)	29 (74.35)	0.53	1.27 (0.6–2.71)
History of thrombosis	47 (30.71)	0.76	0.93 (0.6–1.451)	61 (30.34)	0.89	0.97 (0.63–1.5)	10 (25.64)	0.89	0.97 (0.63–1.5)

Reference categories: Age < 60 years; gender = female; *JAK2*, *CALR* = negative; hemoglobin < 16.5 g/dL; platelets < 450 × 10^9^/L; leukocytes < 11 × 10^9^/L; no exposure to cytoreductive agents; no exposure to noxious substances; spleen normal size; no history of thrombosis; *p*-value < 0.05 was considered significant and is indicated in italics, *n*—number of patients.

## Data Availability

The data that support the findings of this study are available from the corresponding author, [C.B.], upon reasonable request.

## Abbreviations

ALL	Acute lymphoblastic leukemia
AML	Acute myeloid leukemia
*CALR*	Calreticulin
CML	Chronic myeloid leukemia
ET	Essential thrombocythemia
HL	Hodgkin’s Lymphoma
HU	Hydroxyurea
*JAK2*	Janus kinase 2
MAF	Minor Allele Frequency
*MPL*	Myeloproliferative leukemia virus oncogene
MPN	Myeloproliferative neoplasms
NHL	Non-Hodgkin’s Lymphoma
NER	Nucleotide excision repair
PMF	Primary myelofibrosis
PV	Polycythemia vera
PCR-RFLP	Polymerase chain reaction–restriction fragment length polymorphism
SNP	Single nucleotide polymorphism
WHO	World Health Organization
XP	Xeroderma pigmentosum
*XPC*	Xeroderma pigmentosum complementation
UV	Ultraviolet
